# Potential Inhibitory Effect of *Apis mellifera*’s Venom and of Its Two Main Components—Melittin and PLA_2_—on *Escherichia coli* F_1_F_0_-ATPase

**DOI:** 10.3390/antibiotics9110824

**Published:** 2020-11-18

**Authors:** Hala Nehme, Helena Ayde, Dany El Obeid, Jean Marc Sabatier, Ziad Fajloun

**Affiliations:** 1 Bioactive Molecules Research Laboratory, Faculty of Sciences, Section II, Lebanese University, B.P. 90656, Jdeidet El-Matn 1202, Lebanon; helena.ayde@hotmail.com; 2 Faculty of Agriculture & Veterinary Sciences, Lebanese University, Dekwaneh, Beirut 2832, Lebanon; dany.obeid@ul.edu.lb; 3 Institut de Neuro-Physiopathologie, Faculté de Médecine Secteur Nord 51, Université Aix-Marseille, UMR 7051, Boulevard Pierre Dramard-CS80011, 13344-Marseille CEDEX 15, France; 4 Laboratoire de Biotechnologie Appliquée (LBA3B), Centre Azm pour la Recherche en Biotechnologie et ses Applications, EDST, Lebanese University, Tripoli 1300, Lebanon; ziad.fajloun@ul.edu.lb; 5 Department of Biology, Faculty of Sciences, Section III, Lebanese University, Michel Slayman Tripoli Campus, Ras Maska 1352, Lebanon

**Keywords:** antibiotic resistance, F_1_F_0_-ATPase, *Escherichia coli*, *Apis mellifera* bee venom, inhibitory assays, antibacterial activity

## Abstract

Bacterial resistance has become a worrying problem for human health, especially since certain bacterial strains of *Escherichia coli* (*E. coli*) can cause very serious infections. Thus, the search for novel natural inhibitors with new bacterial targets would be crucial to overcome resistance to antibiotics. Here, we evaluate the inhibitory effects of *Apis mellifera* bee venom (BV-*Am*) and of its two main components -melittin and phospholipase A_2_ (PLA_2_)- on *E. coli* F_1_F_0_-ATPase enzyme, a crucial molecular target for the survival of these bacteria. Thus, we optimized a spectrophotometric method to evaluate the enzymatic activity by quantifying the released phosphate from ATP hydrolysis catalyzed by *E. coli* F_1_F_0_-ATPase. The protocol developed for inhibition assays of this enzyme was validated by two reference inhibitors, thymoquinone (IC_50_ = 57.5 μM) and quercetin (IC_50_ = 30 μM). Results showed that BV-*Am* has a dose-dependent inhibitory effect on *E. coli* F_1_F_0_-ATPase with 50% inhibition at 18.43 ± 0.92 μg/mL. Melittin inhibits this enzyme with IC_50_ = 9.03 ± 0.27 µM, emphasizing a more inhibitory effect than the two previous reference inhibitors adopted. Likewise, PLA_2_ inhibits *E. coli* F_1_F_0_-ATPase with a dose-dependent effect (50% inhibition at 2.11 ± 0.11 μg/mL) and its combination with melittin enhanced the inhibition extent of this enzyme. Crude venom and mainly melittin and PLA_2_, inhibit *E. coli* F_1_F_0_-ATPase and could be considered as important candidates for combating resistant bacteria.

## 1. Introduction

Bacteria are microscopic organisms that have a high impact on their surroundings. Most bacteria are harmless and are valuable germs whereas some constitute major threats to public health [1]. The gram-negative *Escherichia coli* (*E. coli*) bacterium, in spite of being a normal member of the intestinal microbiome, is one of the triggers of these outbreaks [2], especially because it has acquired the capacity of resisting usual antimicrobial agents [3]. Thereby, new antibiotics and biological targets are needed to overcome this antibacterial resistance [3].

F_1_F_0_-ATPase is present in all living organisms and located in the membranes of mitochondria, bacteria, and chloroplasts [4]. It is a double-motor enzyme which plays various roles in the cell, participating in adenosine triphosphate (ATP) synthesis and in ATP hydrolysis-dependent processes and in the regulation of a proton gradients across some membrane-dependent systems [5]. Indeed, the F_1_ domain of F_1_F_0_-ATPase, by a clockwise rotation, catalyzes the phosphorylation of ADP to ATP. However, when transmembrane proton motive force (Δp) decreases, F_1_ catalyzes the ATP hydrolysis by a counter clockwise rotation which is accompanied by the transportation of H^+^ back to the intermembrane space [6]. Thus, F_1_F_0_-ATPase is the chief generator of ATP, which is the energy source required to maintain cellular activity and to modulate the signaling pathways in order to ensure cell survival [6,7]. Likewise, this enzyme is emerging as an interesting molecular target for the development of new therapies for a variety of diseases [5,8,9,10].

F_1_F_0_-ATPase is a complicated protein complex. It is divided into two sectors, a soluble globular F_1_ catalytic sector and a membrane-bound F_0_ proton-translocating sector [5]. The F_1_ region is a soluble catalytic hexamer, composed of the alternation of three α and β subunits with the γ subunit inserted in its central slit. The intrinsic part of the F_0_ membrane is hydrophobic and has a ring-shaped c-subunit structure that allows protons to move through the membrane. In fact, the F_1_ sector contains six nucleotide binding sites, three of which are contained in the β subunits and responsible for the hydrolysis and synthesis of ATP [11]. Significantly, the F_1_F_0_-ATPase, because of its complex structure, is inhibited by a number of different inhibitors and provides diverse possibilities in the development of new F_1_F_0_-ATPase-directed agents [5,12]. Indeed, a wide range of natural and synthetic products including polyphenols, peptides, nucleotides and divalent metal ions are known to bind and inhibit F_1_F_0_-ATPase [5,8].

In the past, scientists have become more aware of the health hazards resulting from the consumption of chemical additives and synthetic drugs [13]. Therefore, the new trend is the use of natural treatments at the expense of synthetic ones, such as the use of drugs derived from extracts of plants or animals [14].

Indeed, animal venoms are a cocktail of molecules including peptides, amines and enzymes potentially active on different biological targets of interest. Bee venom is a complex mixture of peptide toxins and enzymes with a wide range of biological activities such as antimicrobial, anticancer and antioxidant effects [15,16]. Bee venom of *Apis mellifera* (BV-*Am*) has been previously analyzed and revealed mainly in the presence of two components, melittin and PLA_2_, which are the most abundant in this venom [15,17]. Other studies have shown that melittin and PLA_2_ contribute generally to the antibacterial activities of the bee venom [18,19]. Hence, the aim of our work is to study the action of BV-*Am,* as well as its two main compounds -melittin and PLA_2_-, on *E. coli* F_1_F_0_-ATPase in order to evaluate their potentially inhibitory effect on this enzyme, revealing consequently the interest for their eventual application as antibacterial substances.

## 2. Results

### 2.1. Optimization of the Phosphate Dosage Method

To measure the inorganic phosphate (P_i_) resulting from ATP hydrolysis during the enzymatic reaction catalyzed by the membrane F_1_F_0_-ATPase, a suitable colorimetric method was chosen as previously described by Lowry et al. 1945 [20] with some optimizations. This P_i_ reacts with the ammonium molybdate to form a phosphomolybdic acid complex which is reduced in the presence of ascorbic acid into a blue molybdous compound that absorbs at 700 nm. This reaction requires an acidic pH [20]. Therefore, ammonium molybdate and ascorbic acid were prepared in H_2_SO_4_ at different concentrations to determine the most suitable one for the dosage simultaneously of high and low concentrations of phosphate. The H_2_SO_4_ concentrations tested were 0.1, 0.25, 0.5 and 1.0 N. Experiments were performed with standard solutions of a low concentration of P_i_ of 5 µM corresponding to the range of values expected to be obtained with inhibitory assays while other assays were performed with standard solutions of a high concentration of P_i_ of 50 µM corresponding to values expected to be reached with enzymatic assays in the absence of an inhibitor.

**Figure 1:**
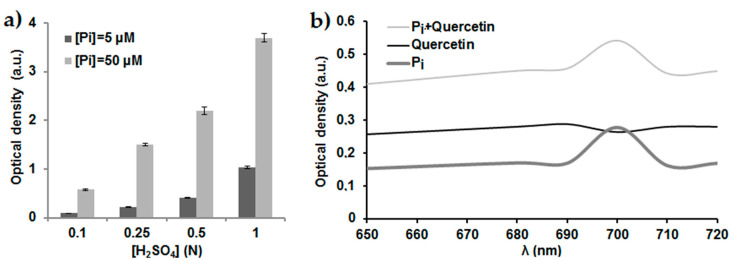
(**a**) Optimization of H_2_SO_4_ concentration for the P_i_ dosage. Standard P_i_ solutions of 5 µM and 50 µM prepared in tris-HCl buffer (pH = 8.5; 50 mM). Incubation time: 10 min. λ = 700 nm. Error bars show the standard deviation obtained from experiments done in triplicate. (**b**) Spectra showing the OD of standard solutions of P_i_, quercetin and a mixture of P_i_ and quercetin. P_i_ = 5 µM; Quercetin = 30 µM.

Our results represented in Figure 1a show that H_2_SO_4_ concentration of 0.1 N was not suitable to our protocol due to low optical density (OD) observed (<0.2) for low concentration of P_i_ of 5 µM. For a concentration of the acid higher than 0.25, OD values observed for the 5 µM P_i_ solution go from 0.21 at 0.25 N H_2_SO_4_ to 1.03 at 1.0 N H_2_SO_4_. These OD values are between 0.2 and 1.5. They are within the linearity domain of the Beer-Lambert law allowing performing quantitative assays [21]. For the highest concentration of 50 µM, the observed OD at 0.1 N and 0.25 N H_2_SO_4_ are 0.57 and 1.49, respectively while OD values observed for H_2_SO_4_ concentrations are higher than 0.25 N and are above 2 and thus deviate from the linearity of the Beer-Lambert law. Hence, the best compromise to achieve quantitative assays throughout this study for low and high P_i_ concentrations was for 0.25 N H_2_SO_4_. Then, a curve was established for different P_i_ standard solutions between 5 and 50 μM to which are added ammonium molybdate and ascorbic acid solutions prepared in 0.25 N H_2_SO_4_. A positive linear correlation is obtained between the OD and the P_i_ (*R*^2^ > 0.9902) with a good repeatability of the measurements since the relative standard deviation (RSD%) is less than 3% for each standard P_i_ solution assayed in triplicate. This regression will be used to quantify the P_i_ released during enzymatic assays catalyzed by the *E. coli* membrane-bound F_1_F_0_-ATPase.

Among the inhibitors tested in this study, quercetin exhibits a yellow color in solution and interferes with the dosage of P_i_ released during the inhibitory assays. Therefore, the spectrum of the colorful complex formed with P_i_ (5 µM) in the presence and absence of quercetin (30 µM) was established between 650 and 720 nm to determine the suitable wavelength for the study (Figure 1b). Results show that 700 nm is suitable for measuring the OD of P_i_ since at this wavelength quercetin has the lowest interference. Indeed, it has the lowest OD (0.26 a.u.) while P_i_ has the highest one (0.28 a.u.). Moreover, since the OD value of quercetin solution at this wavelength is not zero, the OD value observed for a blank solution of a quercetin solution was subtracted from that obtained when quercetin is added to the reaction medium for each inhibitor concentration.

The wavelength of 700 nm was also chosen for the other molecules studied (thymoquinone, melittin and PLA_2_).The blank does not contain the corresponding potential active molecule since it does not give colorful solutions in aqueous media and does not interfere with the dosage of P_i_.

### 2.2. Screening of Natural Components as Potential Inhibitors of E. coli F_1_F_0_-ATPase

**Figure 2:**
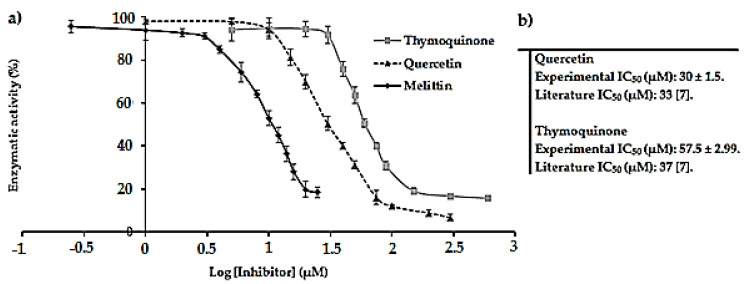
(**a**) Dose-response curve representing the enzymatic activity (%) of *E. coli* F_1_F_0_-ATPase as a function of the log of the concentration of thymoquinone, quercetin and melittin, respectively. Error bars show the standard deviation obtained from experiments done in triplicate; (**b**) Comparison between the IC_50_ values of the reference inhibitors obtained by the developed method and from the literature.

First, the previously optimized method to perform enzymatic assays was verified by testing two reference inhibitors of the *E. coli* F_1_F_0_-ATPase enzyme, namely thymoquinone and quercetin. The dose-response curves representing the enzymatic activity (%) of *E. coli* F_1_F_0_-ATPase in function of log of reference inhibitor concentrations are represented in Figure 2a. The corresponding IC_50_ values obtained are shown in Figure 2b.

The IC_50_ values obtained for quercetin and thymoquinone from the dose-response curves are in good agreement with those documented in the literature. Altogether, these findings validate the optimized method. Good repeatabilities were obtained with relative standard deviations (RSD) on assays below 6% and *R*^2^ values > 0.99.

### 2.3. Study of the Effect of BV-Am on the Membrane-Bound E. coli. F_1_F_0_-ATPase

**Figure 3:**
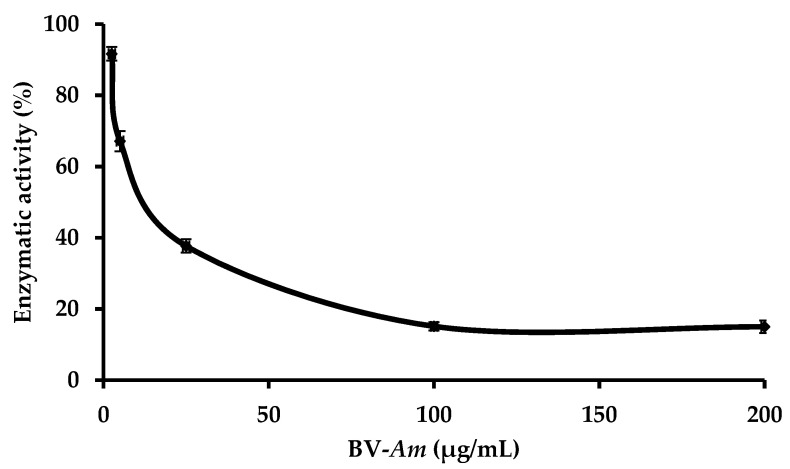
Curve showing the enzymatic activity (%) of the BV-*Am* venom on the membrane-bound *E. coli* F_1_F_0_-ATPase. Error bars show the standard deviation obtained from experiments done in triplicate.

The effect of the BV-*Am* was assayed on the membrane-bound *E. coli*. F_1_F_0_-ATPase. Results obtained are represented in Figure 3 and show that when the BV-*Am* concentration of μM increases, the enzymatic activity decreases until it reaches a plateau. The minimum enzymatic activity reached is 15% while 50% inhibition of the enzyme is observed at 18.43 ± 0.92 µg/mL of the BV-*Am*.

### 2.4. Study of the Inhibition of the E. coli F_1_F_0_-ATPase by PLA_2_

**Figure 4:**
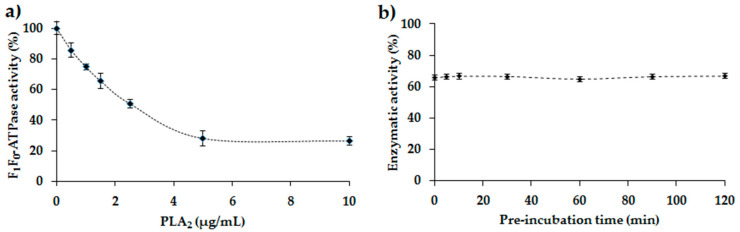
(**a**) Curve showing the effect of different concentrations of PLA_2_ on the membrane-bound *E. coli* F_1_F_0_-ATPase. Error bars show the standard deviation obtained from experiments done in triplicate. Incubation time: 10 min; (**b**) Graph showing the activity of membrane-bound *E. coli* F_1_F_0_-ATPase as a function of different pre-incubation times of the enzyme in presence of the PLA_2_ before the addition of ATP. (ATP) = 100 µM. (PLA_2_) = 1.5 µg/mL. Error bars show the standard deviation obtained from experiments done in triplicate.

Different concentrations of PLA_2_ were tested to determine the maximum percentage inhibition of membrane-bound *E. coli* F_1_F_0_-ATPase (Figure 4a). This inhibition starts linear but then reaches a plateau. Maximum inhibition is around 75% (minimum enzymatic activity 25%) while 50% inhibition is observed at 2.11 ± 0.11 µg/mL PLA_2_.

Several pre-incubation times between 5 min and 2 h were tested to determine the effect of pre-incubation of the membrane-bound *E. coli* F_1_F_0_-ATPase with PLA_2_ before the addition of the substrate ATP (Figure 4b). Results obtained show no significant difference in F_1_F_0_-ATPase activity for different pre-incubation times. In fact, the enzymatic activity observed is approximately 65% at the various pre-incubation times. Thus, for rapid enzymatic analyses, no pre-incubation was set for the rest of the tests carried out in the presence of PLA_2_.

### 2.5. Inhibition of E. coli F_1_F_0_-ATPase by Melittin: Determination of the IC_50_ Value

The IC_50_ value of melittin determined from the dose-response curve is equal to 9.03 ± 0.27 µM (Figure 2a) and is in good agreement with that reported in the literature (7.84 µM) [8]. Good repeatabilities were obtained with relative standard deviations (RSD) below 6% and *R*^2^ > 0.99. Likewise, melittin is found to be a strong potent inhibitor of *E. coli* F_1_F_0_-ATPase with its low IC_50_ value in the micromolar range.

### 2.6. Determination of the Kinetic Parameters of the Enzymatic Reaction Catalyzed by F_1_F_0_-ATPase in the Absence (K_m_ and V_max_) and in the Presence of Melittin (K′_m_ and V′_max_)

**Figure 5:**
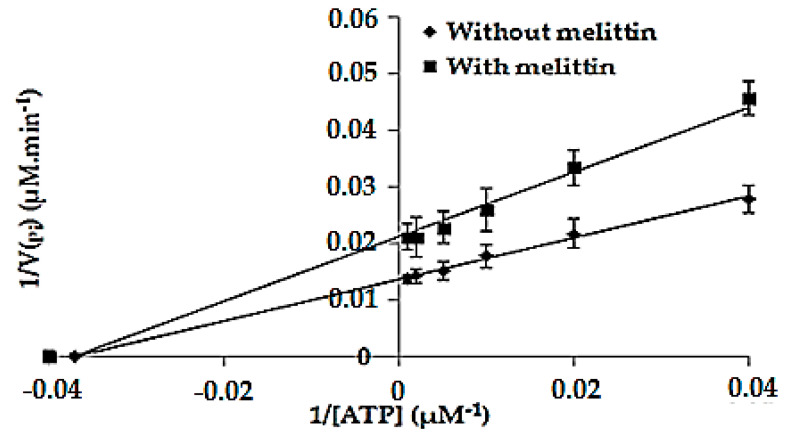
Lineweaver-Burk representation. The points of intersection with the *x*-axis of the linear regressions obtained without and with melittin correspond to −1/K_m_ and −1/K′_m_, respectively while the points of intersection of these lines with the y-axes correspond to 1/V_max_ and 1/V′_max_, respectively. Error bars show the standard deviation obtained from experiments done in triplicate.

The kinetic parameters K_m_ and V_max_ of the enzymatic reaction catalyzed by the membrane-bound *E. coli* F_1_F_0_-ATPase were determined in the optimal conditions. For that matter, the rate of the product formation (V_Pi_) was determined at different concentrations of ATP (Figure 5). K_m_ and V_max_ values obtained in the absence of melittin from the Lineweaver–Burk representation were 26.87 ± 1.34 µM and 72.99 ± 3.67 µM.min^−1^, respectively, while K′_m_ and V′_max_ values obtained in presence of melittin were 26.76 ± 1.32 µM and 46.95 ± 2.34 µM.min^−1^, respectively. The relative standard deviation (RSD%) was less than 5%, indicating a good repeatability of the measurements. Furthermore, the protein amount in the samples was 0.43 ± 0.21 mg/mL, thus the specific activity of the enzyme was about 1.64 μM/min/mg. These results show that melittin is a non-competitive inhibitor of the membrane-bound *E. coli* F_1_F_0_-ATPase since K_m_ remained unchanged (K_m_ = K′_m_) while causing a decrease in the maximum rate from 72.99 to 46.95 µM × min^−1^.

### 2.7. Combination Effect between Melittin and PLA_2_

**Figure 6:**
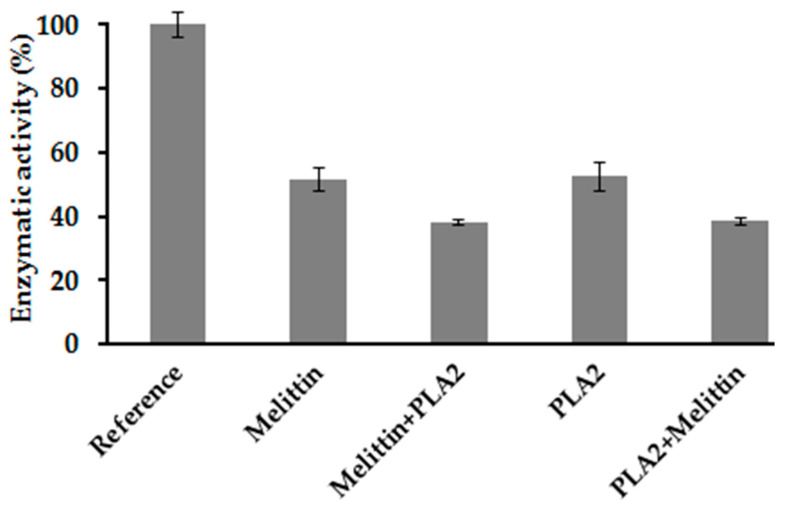
Synergistic action of melittin and PLA_2_ on the membrane *E. coli* F_1_F_0_-ATPase. Reference shows maximal enzymatic activity (100%) obtained for an enzymatic reaction in optimal conditions and in the absence of inhibitor. (ATP) = 100 µM, (Melittin) = 9 µM, and (PLA_2_) = 2.5 µg/mL. Error bars show the standard deviation obtained from experiments done in triplicate.

The combination action of PLA_2_ and melittin on the membrane-bound *E. coli* F_1_F_0_-ATPase was studied by performing assays on the enzyme in the presence of melittin alone and PLA_2_ alone and then mixed together. Results obtained are represented in Figure 6.

The tests carried out show a remaining enzymatic activity of 51.7% in the presence of melittin alone at a concentration of 9 μM corresponding to its IC_50_ value, while the enzymatic activity in the presence of PLA_2_ alone at 2.5 μg/mL is 52.4%. When the membrane-bound *E. coli* F_1_F_0_-ATPase is pre-incubated in the presence of melittin before the addition of PLA_2_ and ATP (Figure 6: melittin + PLA_2_), additional inhibition is observed since the remaining enzymatic activity is 38.1%. The same effect is obtained when the enzyme is pre-incubated with PLA_2_ before adding melittin and ATP (Figure 6: PLA_2_ + melittin), with an enzymatic activity in this case of 38.5%. Hence, results show that the order of addition of these inhibiting agents does not affect their effect since the remaining enzymatic activity is of the same order of magnitude (38%). Good repeatabilities were obtained with relative standard deviations (RSD) below 5%.

To determine the relation of activity between melittin and PLA_2_, the combination index (*CI*) was calculated using Equation (1):

(1) $$CI\;=\;\frac{9}{17}+\frac{2.5}{5.6}\;=\;0.98$$

The *CI* value obtained is below 1. The Student’s *t*-test is performed and *t_c_* value was calculated using Equation (2) as follows:

(2) $$t_{c}=\frac{\left|0.98-1\right|}{0.045/\sqrt{3}}=0.77$$.

The theoretical value (*t_th_*) was 2.776, obtained for (*n* − 3) degrees of freedom and *p* = 0.05. Hence, *t_c_* < *t_th_*.

## 3. Discussion

The resistance of bacteria to the usual antibiotics has become a worrying problem [2,3] since they lead to very serious infections in humans [2,22]. Thus, the search for new extracted natural bioactive molecules and new therapeutic targets is the center of interest for researchers [3,23,24]. The interest of this work is to study the inhibitory effect of the BV-*Am* on the membrane-bound *E. coli* F_1_F_0_-ATPase and of its two main components—melittin and PLA_2_—using a spectrophotometric method that has been optimized for this purpose. In fact, the developed method starting from the isolation of the membrane-bound *E. coli* F_1_F_0_-ATPase to the quantification of the released phosphate (P_i_) using spectrophotometry is a simple yet effective protocol. Indeed, it is economic and requires no sophisticated equipment or reagents to perform assays.

This work aims also to highlight the presence of bioactive molecules in the BV-*Am* venom and to better understand their mechanism of action.

*Apis mellifera* bee venom is a complex mixture of peptide toxins, enzymes and other trace components, with a wide range of biological activities such as antimicrobial, anticancer and antioxidant activities. It has been used as a therapeutic tool in oriental medicine to treat several human inflammatory diseases such as rheumatism, arthritis, and back pain relief [15]. Previously, BV-*Am* has been shown to be active against *Pseudococcus aeruginosa* and *Staphylococcus aureus* [15]. However, to our knowledge, there are no assays performed to study the inhibitory effect of this venom on the membrane-bound *E. coli* F_1_F_0_-ATPAse. Moreover, other previous studies have reported that melittin and PLA_2_ are probably the main components of bee venom, which exhibit its potential activity [15,17]. Therefore, the effect of the BV-*Am* and of its two main components—melittin and PLA_2_—on this enzyme was investigated.

To our knowledge, the effect of PLA_2_ of bee venom on the membrane-bound *E. coli* F_1_F_0_-ATPAse was not yet studied in the literature. However, one study shows that PLA_2_ from snake venom (belonging to the same family of PLA_2_ secreted or sPLA_2_-like bee venom PLA_2_) increased the activity of H^+^-ATPase plasma membrane of maize root cells during the first 15 s, while it inhibits its activity after a longer exposition [25]. This study is partially correlated with our results obtained concerning the effect of PLA_2_ on *E. coli* F_1_F_0_-ATPAse. Thus, we show that PLA_2_ from bee venom inhibits the membrane-bound *E. coli* F_1_F_0_-ATPAse with a maximal inhibitory effect of 75% whatever the duration of pre-incubation of the enzyme with the PLA_2_ was before adding ATP, the substrate of the enzymatic reaction. This constant inhibitory effect is probably due to the fact that the study was performed on the isolated membrane-bound *E. coli* F_1_F_0_-ATPase and not on total bacterial cell.

Melittin is one of the main components of bee venom. Its inhibitory effect on the membrane-bound *E. coli* F_1_F_0_-ATPase was studied in this work and the obtained results are in good agreement with those previously reported in the literature [8,10,26]. Indeed, a maximum inhibitory effect of about 80% was obtained with an IC_50_ value of 9.03 µM (Figure 2a) showing that melittin is an efficient inhibitor of the enzyme on the micromolar scale. This IC_50_ value shows that this molecule inhibits the enzyme with a higher affinity than that observed for the two reference inhibitors assayed in the membrane-bound *E. coli* F_1_F_0_-ATPase [8]. Thus, we can suggest considering melittin from animal venoms as a new reference inhibitor, which could be also used for the study of *E. coli* F_1_F_0_-ATPase. Moreover, melittin was found to be a non-competitive inhibitor of the F_1_F_0_-ATPase (K′_m_ = K_m_ and V′_max_ ≠ V_max_), which means that it binds to a different site than that of ATP on the enzyme. Indeed, it was reported in the literature that melittin binds to the residues 380–386 of the F_1_ sector called the βDELSEED loop of the enzyme, leading to its inhibition [8,9,27].

Henceforth, we show that the BV-*Am* exerts an inhibitory effect on the *E. coli* F_1_F_0_-ATPase enzyme but that melittin is not the only bioactive component of the venom. Indeed, PLA_2_, which is also a component of the bee venom, has an inhibitory effect on the enzyme. Furthermore, other previous studies performed on whole bacterial cells in the literature have reported that melittin activates PLA_2_ by exposing the membrane phospholipids to the catalytic site of enzymes [17]. However, our work shows no significant difference between the calculated CI value (0.98) and 1 since *t_c_* < *t_th_* which reveals an additive effect of PLA_2_ and melittin on the isolated membrane-bound F_1_F_0_-ATPase. This additivity explains the enhanced inhibitory effect observed by the BV-*Am* crude on the F_1_F_0_-ATPase. Our results explain the additional inhibitory effect exhibited by the BV-*Am* shown previously on the membrane-bound *E. coli* F_1_F_0_-ATPase compared to the standards applied to each alone. Moreover, this result is in agreement with the literature where the possible additive effect of some peptides on E. coli ATPase was demonstrated [28].

Even though the results obtained for BV-*Am* show the inhibitory effect of melittin and PLA_2_, fractionation of the venom was performed in order to assay each fraction alone on the membrane-bound F_1_F_0_-ATPase to check the presence of any other enzymes, proteins or peptides bioactive in the medium [26,29].

This work highlights the presence of natural bioactive molecules in BV-*Am* as new sources of novel antibiotics. Further studies on other venom from other types of bees, snakes, scorpions, spiders, etc., can be performed for the same purpose of finding new efficient antimicrobial agents to overcome bacterial resistance. Moreover, identification and characterization of efficient inhibitors of F_1_F_0_-ATPase is highly important since the switching role of the F_1_F_0_-ATPase as a key between life and cell death is increasingly emphasized [30,31,32]. Indeed, F_1_F_0_-ATPase is not only involved in bacterial infections, but studies have shown its implication in many cellular processes such as lipid metabolism [5], angiogenesis [9], inflammation [33] and other pathological conditions such as cardiovascular diseases [34], neurodegenerative conditions [35], obesity [5], type 2 diabetes [36], aging and dementia [37] and in the regulation of energy metabolism [38]. In addition, it is a relevant target for inhibiting oncogenic transformation and tumor cell proliferation by integrating the bioenergetic and death-signaling functions of mitochondria [10,39]. Moreover, F_1_F_0_-ATPase is an important enzyme involved in the mitochondrial permeability transition pore (PTP), the opening of which triggers cell death [40,41]. It is also a primary hub to mitohormesis by activating reactive oxygen species (ROS)-dependent signaling pathways that promote cell survival [6,42]. Thus, F_1_F_0_-ATPase constitutes an attractive target for the treatment of several human diseases [5,31,32].

## 4. Materials and Methods

### 4.1. Venom

The venom was collected from healthy bee colonies of *Apis mellifera*. The collection was carried out using the electroshock method [24].The dried venom was stored in the laboratory at −20 °C until further analysis. Extraction was carried out for 15–20 min on each colony and was repeated twice every two weeks.

### 4.2. Reagents and Chemicals

Adenosine 5′-triphosphate disodium salt hydrate ATP (99%), ammonium molybdate tetrahydrate (99%), dimethyl sulfoxide DMSO (99.9%), ethylenediaminetetraacetic acid disodium salt dehydrate EDTA (99%), potassium phosphate monobasic KH_2_PO_4_, and Trizma base (99%) were purchased from Sigma-Aldrich (St Louis, MO, USA). L-Ascorbic acid (99%) was provided by HiMedia (Mumbai, India). Magnesium chloride MgCl_2_ was furnished by Acros organics (New Jersey, NJ, USA). Hydrochloric acid HCl and sulfuric acid H_2_SO_4_ (96.3%) were both supplied by VWR chemicals. Glycerol was purchased from Loba Chemie (Mumbai, India). The natural molecules quercetin (95%) and thymoquinone (99%) were purchased from Sigma-Aldrich (St Louis, MO, USA). PLA_2_ and melittin from the *Apis mellifera* bee were purchased from Latoxan Laboratory (France).

### 4.3. Sources of Bacterial Strain and Culture Media

*Escherichia coli* (ATCC^®^ 25922™) was acquired from the American type culture collection ATCC (Manassas, VA, USA). This strain is classified as biosafety level 1(BSL-1) since it is a nonpathogenic strain. Nutrient Broth and Nutrient Agar were both procured from CONDA (Madrid, Spain).

### 4.4. Bacterial Cultures

Two to three colonies of *E. coli* are dispersed in 5 mL of nutrient medium (TSB). Then, the inoculated medium is incubated for 18 h at 37 °C, with the caps left slightly open. The next day, the optical density (OD) of the preculture is measured at 620 nm using a UV-VIS spectrophotometer and this value corresponds to the number of bacteria multiplied by 10^9^(CFU/mL).

### 4.5. Preparation of the Bacterial Sample

Isolation of the membrane-bound F_1_F_0_-ATPase was performed as described by Issa et al. Ref. [7] with minor modifications. Briefly, 500 μL of the bacterial suspension are centrifuged for 15 min at 4 °C and 10,000× *g* to remove the nutrient medium (TSB). The pellet is then resuspended in a tris-HCl buffer (50 mM; pH 8.5) containing 25% (*v/v*) of EDTA in a final volume of 400 μL and incubated for 10 min at room temperature. Next, 100 μL of glycerol are added with a final concentration of 4% (*v/v*) to the bacterial suspensions before subjecting the samples to a freeze-thaw cycle followed by three cycles of sonication on ice for one minute each. Then a second centrifugation for 15 min at 4 °C at 10,000× *g* is applied to obtain the pellet containing the isolated membrane-bound F_1_F_0_-ATPase. The supernatant is then removed. The pellet is washed twice with tris-HCl buffer (50 mM, pH 8.5) to remove glycerol and EDTA. Finally, the isolated membrane-bound F_1_F_0_-ATPase contained in the pellet is ready to be assayed.

### 4.6. Phosphate Quantification Protocol

F_1_F_0_-ATPase catalyzes either the formation of ATP from adenosine diphosphate (ADP) and inorganic phosphate (P_i_) or the opposite reaction. In this study, ATP hydrolysis is investigated and the reaction is evaluated by quantifying the P_i_ produced. The concentration of P_i_ is deduced from a linear regression obtained from different concentrations of standard P_i_ solutions ranging between 0.5 and 100 μmol/L. Standard solutions are prepared by serial dilutions of a stock solution of phosphate in tris-HCl (50 mM, pH 8.5) buffer. The P_i_ dosage is determined according to the method of Lowry et al. [20] with minor modifications. Briefly, 100 μL of a 1% solution of ammonium molybdate prepared in H_2_SO_4_ is mixed with 1000 μL P_i_ solutions leading to the formation of a phosphomolybdic acid complex. The mixture is then incubated for 10 min at room temperature with 100 μL of a 1% ascorbic acid solution prepared in H_2_SO_4_ leading to a blue molybdous compound. The optical density values of the complexes are measured by a spectrophotometer at 700 nm. A blank solution of tris-HCl (50 mM, pH 8.5) undergoes the same treatment. Assays for each phosphate concentration were done in triplicate.

### 4.7. Study of the Inhibition of E. coli F_1_F_0_-ATPase

In this work, two natural components were tested as two known inhibitors against F_1_F_0_-ATPase from *E. coli* [7]. Inhibitory assays were performed as follows: the isolated membrane-bound F_1_F_0_-ATPase, obtained as described in Section 4.5, is resuspended in appropriate volumes of tris-HCl buffer (50 mM; pH 8.5) and MgCl_2_ (15 mM) before incubation for 10 min at 37 °C. Then, different volumes of stock solution of reference inhibitors are added to the medium followed by ATP at 100 μM (saturating concentration of ATP). The samples are incubated for 40 min at 37 °C. Then the reaction is stopped by adding 1% (*v/v*) SDS. For inhibitory assays performed in the presence of thymoquinone, a blank tube is treated in the same way without adding the inhibitor or ATP to the suspension. For inhibitory assays in the presence of quercetin, a blank is prepared for each quercetin concentration tested and treated as for the inhibitory assays without adding ATP. To determine the maximum activity of the enzyme, a test is carried out in the absence of an inhibitor. In this case, the percentage of enzymatic activity is considered to be 100%. Then, the pellet is removed after centrifugation (10 min, 10,000× *g*) and the P_i_ present in the supernatant is assayed as indicated in Section 4.6 to determine the enzymatic inhibition. All tests were performed in triplicate.

### 4.8. Determination of IC_50_ Values of References and Potential Inhibitors of Membrane-Bound E. coli F_1_F_0_-ATPase

In order to calculate the inhibitory concentration (IC_50_) [43], which corresponds to the concentration of the inhibitor required to exert 50% inhibition of maximal enzyme activity [43], inhibitory assays were performed as described in Section 4.6. IC_50_ values were determined using the PRISM^®^ 5.04 (GraphPad software, San Diego, CA, USA) software, from a dose-response curve established by drawing the percentage of enzymatic activity (%) at different concentrations of the inhibitor expressed in terms of a log (inhibitor) (μM). The inhibitor is reckoned to be more active as much as its IC_50_ value is low [44].

### 4.9. Study of the Potential Inhibitory Effect of BV-Am on the Membrane-Bound E. coli F_1_F_0_-ATPase

The effect of the BV-*Am* on the membrane-bound *E. coli* F_1_F_0_-ATPase is studied as described in Section 4.6. Different volumes of the stock solution of venom prepared in the buffer tris-HCl (pH = 8.5; 50 mM) are added to the reaction medium containing the transmembrane enzyme followed by ATP at 100 μM. The samples are incubated for 40 min at 37 °C. The reaction is then stopped by the addition of 1% SDS (*v/v*). The percentage of enzymatic activity (%) as a function of different concentrations of the venom (0–200 µg/mL) is presented. All assays were performed in triplicate.

### 4.10. Study of the Potential Inhibitory Effect of PLA_2_ on the Membrane-Bound E. coli F_1_F_0_-ATPase

The effect of PLA_2_ on the membrane-bound F_1_F_0_-ATPase is studied as described in Section 4.6 with some modifications. Different volumes of the stock solution of phospholipase A_2_ are added to the reaction medium containing the isolated membrane-bound enzyme followed by ATP at 100 μM. The samples are incubated for 40 min at 37 °C. The percentage of enzymatic activity (%) in the function of different concentrations of PLA_2_ (0–10 µg/mL) is presented.

The effect of pre-incubation on the membrane-bound F_1_F_0_-ATPase in the presence of PLA_2_ before adding ATP is also studied. Different pre-incubation times from 0 to 120 min before adding ATP were tested. All assays were performed in triplicate.

### 4.11. Study of the Potential Inhibitory Effect of Melittin on the Membrane-Bound E. coli F_1_F_0_-ATPase

The effect of melittin on the membrane-bound F_1_F_0_-ATPase is studied as described in Section 4.7. Different volumes of the stock solution of melittin are added to the reaction medium containing the enzyme followed by ATP at 100 μM. The samples are incubated for 40 min at 37 °C. The reaction is then stopped using a solution of 1% SDS (*v/v*). A blank tube is treated in the same way without adding melittin or ATP to the suspension. IC_50_ values were obtained from the dose-response curve using PRISM^®^ 5.04 software. All assays were done in triplicate.

In order to determine the type of inhibition exerted by melittin on the enzyme (competitive, non-competitive), enzymatic assays were performed as described in Section 4.7. with some modifications. Briefly, a first series of assays is performed by incubating the isolated membrane-bound *E. coli* F_1_F_0_-ATPase with different concentrations of ATP ranging from 0 to 1000 µM without melittin, while in the second series, a fixed concentration of melittin corresponding to its IC_50_ value is added before introducing ATP to the mixture. After incubation for 40 min at 37 °C, the reaction is stopped by adding SDS (1%). The P_i_ released from the enzymatic reaction is calculated from the linear regression as described in Section 4.6. Then, kinetic parameters of the enzymatic reaction are determined from the Lineweaver-Burk representation by plotting 1/V_Pi_ as a function of 1/(ATP); V_Pi_ is the rate of formation of the product (P_i_) which corresponds to the ratio of the concentration of the P_i_ on the incubation time (40 min). K_m_, V_max_, K′_m_ and V′_max_ were determined by fitting results on PRISM^®^ 5.04. All assays were performed in triplicate.

### 4.12. Study of the Effect of Combination of PLA_2_ and Melittin on the Membrane-Bound E. coli F_1_F_0_-ATPase

To determine the combination effect between melittin and PLA_2_ on *E. coli* membrane-bound F_1_F_0_-ATPase, assays were performed as described in Section 4.7. with some modifications. In fact, assays were performed by adding to the medium before the addition of ATP at 100 µM, melittin at 9 µM alone, PLA_2_ at 2.5 µg/mL alone, melittin followed by PLA_2_ or PLA_2_ followed by melittin before adding ATP. The combination index (CI) was then calculated as follows [45]:

(3) $$CI\;\;=\;\;\frac{A_{c}}{A_{E}}+\frac{B_{c}}{B_{E}}$$

where *A_c_* and *B_c_* are respectively the concentrations of melittin and PLA_2_ in the mixture, and *A_E_* and *B_E_* are respectively the concentrations of single melittin and PLA_2_ capable of producing the same effect as in the mixture. If *CI* < 1, a synergy action is demonstrated between A and B. If *CI* = 1, an additive effect is shown between A and B. If *CI* > 1, an antagonistic effect between A and B is confirmed.

### 4.13. Statistical Analysis

Results were expressed as the mean ± standard deviation (SD). Statistical significance between different samples was analyzed using a two-tailed unpaired *t*-test. This analysis was carried out using GraphPad Prism 5.04 (GraphPad Software, San Diego, CA, USA). Statistical significance was defined as *p* < 0.05.

The calculated *CI* was compared to the reference value (*µ* = 1) to evaluate if the difference is significant using the Student’s *t*-test:

(4) $$t_{c}=\frac{\left|CI-\mu \right|}{s/\sqrt{n}}$$

where *CI*, *s* and *n* correspond to the *CI* value, the standard deviation and the number of assays performed, respectively.

*t_c_* was compared to the theoretical value(*t_th_*) determined from the t-table for *n* − 1 and α = 5%.
